# Robbing Peter to Pay Paul: Chlorhexidine gluconate demonstrates short‐term efficacy and long‐term cytotoxicity

**DOI:** 10.1111/wrr.13044

**Published:** 2022-08-11

**Authors:** J. Z. Alex Cheong, Aiping Liu, Clayton J. Rust, Collin L. Tran, Sameeha E. Hassan, Lindsay R. Kalan, Angela L. F. Gibson

**Affiliations:** ^1^ Department of Medical Microbiology and Immunology University of Wisconsin–Madison, School of Medicine and Public Health Madison Wisconsin USA; ^2^ Microbiology Doctoral Training Program University of Wisconsin–Madison Madison Wisconsin USA; ^3^ Department of Surgery University of Wisconsin–Madison, School of Medicine and Public Health Madison Wisconsin USA; ^4^ Department of Medicine, Division of Infectious Disease University of Wisconsin–Madison, School of Medicine and Public Health Madison Wisconsin USA

**Keywords:** antimicrobial efficacy, antiseptics, chlorhexidine gluconate, cytotoxicity, ex vivo human skin, wound cleansing

## Abstract

Wound cleansing agents are routine in wound care and preoperative preparation. Antiseptic activity intends to prevent contaminating microbes from establishing an infection while also raising concerns of cytotoxicity and delayed wound healing. We evaluated the cytotoxicity of five clinically used wound cleaning agents (saline, povidone iodine, Dove® and Dial® soaps, and chlorhexidine gluconate [CHG]) using both an ex vivo and in vivo human skin xenograft mouse model, in contrast to classical in vitro models that lack the structural and compositional heterogeneity of human skin. We further established an ex vivo wound contamination model inoculated with ~100 cells of *Pseudomonas aeruginosa* or *Staphylococcus aureus* to evaluate antimicrobial efficacy. Scanning electron microscopy and confocal microscopy were used to evaluate phenotypic and spatial characteristics of bacterial cells in wound tissue. CHG significantly reduced metabolic activity of the skin explants, while all treatments except saline affected local cellular viability. CHG cytotoxicity persisted and progressed over 14 days, impairing wound healing in vivo. Within the contamination model, CHG treatment resulted in a significant reduction of *P. aeruginosa* wound surface counts at 24 h post‐treatment. However, this effect was transient and serial application of CHG had no effect on both *P. aeruginosa* or *S. aureus* microbial growth. Microscopy revealed that viable cells of *P. aeruginosa* reside deep within wound tissue post‐CHG application, likely serving as a reservoir to re‐populate the tissue to a high bioburden. We reveal concerning cytotoxicity and limited antimicrobial activity of CHG in human skin using clinically relevant models, with the ability to resolve spatial localization and temporal dynamics of tissue viability and microbial growth.

**Abbreviations**
CFU: colony forming unit
CHG: chlorhexidine gluconate
CLSM: confocal laser scanning microscopy
DMEM: Dulbecco's modified Eagle's medium
FBS: fetal bovine serum
GFP: green fluorescent protein
hpi: hours post‐inoculation
hpt: hours post‐treatment
LDH: lactate dehydrogenase
PBS: phosphate‐buffered saline
PVI: povidone iodine
SEM: scanning electron microscopy

## 1 INTRODUCTION

During routine wound care, control of microbial colonization and bioburden is critical to aid tissue repair and reduce excess inflammation.^1^, ^2^, ^3^ Wound cleansing with agents ranging from saline to solutions of antiseptic compounds are routinely employed in an effort to prevent microbial colonization and subsequent infection of wound tissue.^4^, ^5^, ^6^ A major consideration in the type of cleansing agent used is antimicrobial efficacy, however antiseptics with high antimicrobial activity also exhibit considerable cytotoxicity and may further inhibit tissue repair.^7^, ^8^, ^9^, ^10^, ^11^ Current guidelines from the World Health Organization recommend preoperative skin preparation with CHG,^12^ however recent studies question the superiority of CHG in preventing surgical site infections.^13^, ^14^

Classical models used to evaluate antiseptic efficacy and cytotoxicity use reductionist approaches that evaluate components of human skin and individual infectious microbes in silos, for example, through the use of pure cell culture and bacterial culture in vitro.^15^, ^16^, ^17^, ^18^, ^19^, ^20^, ^21^, ^22^, ^23^, ^24^, ^25^, ^26^, ^27^, ^28^ Importantly, such models lack the structural and compositional heterogeneity of human skin and spatiotemporal nature of wound tissue. In vitro models are thus unsuitable to concurrently evaluate the antimicrobial efficacy and cytotoxicity of antiseptic agents under clinically relevant scenarios.^29^ There is a critical need for clinically applicable models that can simultaneously evaluate antimicrobial efficacy and cytotoxicity of antimicrobial interventions under relevant contexts.^30^, ^31^, ^32^

Here, we use an ex vivo human skin excisional wound model to evaluate wound cleaning agents routinely used in standard of care as a prophylaxis for infection. To evaluate the antimicrobial efficacy, we established a wound contamination model by inoculating excisional wounds with ~100 cells of either *Pseudomonas aeruginosa* or *Staphylococcus aureus* and evaluated the bioburden over time after topical application of wound cleansing agents. To concurrently evaluate spatial and temporal dynamics, we use histological staining of cellular viability via lactate dehydrogenase (LDH) activity and microscopy to localize cytotoxicity and microbial colonization of the wound bed. We find that CHG exhibits the greatest level of cytotoxicity and this is associated with greater antimicrobial efficacy early after application. However, at later timepoints we show that CHG is ineffective against *P. aeruginosa* and *S. aureus*, even with serial application. Further, we demonstrate that CHG cytotoxicity persists over time and inhibits wound healing in an in vivo human skin xenograft mouse model.

## 2 MATERIALS AND METHODS

### 2.1 Ex vivo excisional wound model

Human skin was obtained from patients undergoing elective reconstructive surgeries. The de‐identified samples were exempt from the regulation of University of Wisconsin‐Madison Human Subjects Committee Institutional Review Boards. The tissue was rinsed with PBS and partial thickness wounds were made by lightly puncturing the epidermis with a 6 mm biopsy punch and removing the entire epidermis and a portion of the dermis with scissors. While donor‐to‐donor variability in skin thickness is expected, the wounding for each biological replicate was conducted by one investigator to avoid inter‐personal variation. A 12 mm biopsy punch was then used to make full‐thickness biopsies with the wound. In the antiseptic antimicrobial efficacy studies, biopsies were placed into 12‐well plates containing 3 ml of a DMEM‐agarose gel (0.15:0.85 ratio of 1% agarose in PBS and DMEM [Gibco, Thermo Fisher, Waltham, MA] supplemented with 10% fetal bovine serum [FBS; Gibco, Thermo Fisher, Waltham, MA]). In the antiseptic cell viability studies, biopsies were placed onto a fine mesh insert in p100 plates to raise the tissue to the air–liquid interface in media containing 10 ml of DMEM supplemented with 10% FBS, 0.625 μg/ml amphotericin B (Thermo Fisher, Waltham, MA), and 100 μg/ml of penicillin‐streptomycin (Thermo Fisher, Waltham, MA). Biopsies were incubated at 37°C with 5% CO_2_ and were transferred to a new medium every 48 h. Experiments were conducted within 48 h of tissue collection with the exception of *S. aureus* inoculations, where biopsies were incubated for 5 days (2 media passages) before inoculation to wash out residual patient antibiotics.

### 2.2 Ex vivo wound colonization model

*Pseudomonas aeruginosa* strain K (gift from Dr. Anna Huttenlocher, University of Wisconsin–Madison) or *Staphylococcus aureus* strain LAC (gift from Dr. JD Sauer, University of Wisconsin–Madison; both GFP‐tagged with carbenicillin selection) were grown overnight at 37°C on tryptone soy agar (TSA) plates supplemented with 200 μg/ml carbenicillin. Inoculums were prepared by suspending colonies from agar plates into PBS and diluting to a cell density of 1 × 10^4^ CFU/ml. Wounds were inoculated with 10 μl of inoculum for a final cell density of 1 × 10^2^ CFU/wound. Following 4 h of incubation, wounds were treated with antiseptics (see below), rinsed, incubated for 24 h, and then processed for microscopy (see below) or bisected and processed for viable cell enumeration. A subset of PBS‐ and CHG‐treated biopsies was immediately processed after treatment at 4 h post‐inoculation, or treated for a second time at 24 h post‐treatment and incubated for 24 h before processing. All bisects were vortexed in 1 ml PBS with 5% Tween 80 and 0.6% sodium oleate (as CHG neutralizer) with 0.2 g of 1 mm sterile glass beads for 10 min at full‐speed on a Vortex‐Genie 2 (Scientific Industries, Bohemia, NY) before serial dilution and spot plating 20 μl on TSA plates with no antibiotic supplementation.

### 2.3 Antiseptic treatment

Ex vivo wound tissue biopsies were treated with five antiseptics including PBS (Corning, Corning, NY), povidone iodine (PVI; Medichoice, Owens and Minor, VA), Dial® (Henkel, Scottsdale, AZ) and Dove® soaps (Unilever, London, UK), and CHG (2%, BD, Franklin Lakes, NJ). Dial® and Dove® soaps were diluted 1:1 in sterile water 24 h before treatment and allowed to mix. Wound cleansing solutions were applied onto the wound by gently blotting with sterile cotton swabs (Medline, Northfield, IL) around the wound edge until the solution pooled on the wound bed. The treatment was left on for 30 min before gently rinsing twice with PBS. For cell viability studies, the biopsies were incubated for an additional 24 h before the MTT assay and LDH staining (see below) were performed. To determine whether the cytotoxicity induced by CHG persists over time, a subset of PBS‐ and CHG‐treated biopsies were cultured at 37°C and 5% CO_2_ for up to 14 days. Tissues were harvested on day 1, 3, 7, and 14 and processed for LDH staining. To mimic in vivo treatment parameters with this model, biopsies were treated daily with PBS or CHG and harvested on day 14 for the MTT assay and LDH staining.

### 2.4 Minimum inhibitory concentration testing

*P. aeruginosa* strain K and *S. aureus* strain LAC were grown overnight at 37°C on TSA plates. Inoculums were prepared by suspending colonies from agar plates into PBS and diluting to a final cell density of 1 × 10^5^ CFU/ml in tryptone soy broth for broth microdilution with CHG from concentrations of 1000 μg/ml to 2 μg/ml. The minimum inhibitory concentration (MIC) was interpreted as the lowest concentration of CHG that inhibited visual growth.

### 2.5 Tissue metabolic activity assay

At 24 h post‐treatment, cell viability of treated tissues was quantified using a tetrazolium‐based (MTT) assay.^33^ Briefly, each bisect was rinsed in PBS and placed in an individual well of a 6‐well plate with 2 ml MTT solution (2 mg/ml, Invitrogen, Thermo Fisher, Waltham, MA) in each well. The 6‐well plates were placed on a rotating plate and incubated at 37°C at 100 rpm for 2 h. After aspiration of the remaining MTT solution, 4 ml DMSO was added to each well and incubated at 100 rpm at 37°C for 80 min. Exactly 200 μl aliquots of solution in each well were transferred to a 96‐well plate with DMSO blank controls. The optical density of the solution was measured using a plate reader (FlexStation 3, Molecular Devices, San Jose, CA) at a wavelength of 540 nm. For the MTT assay, untreated tissue biopsies were immersed in boiling water for 30 min as a negative control, while the PBS‐treated biopsies served as the positive control.

### 2.6 Histological tissue processing

Tissue bisects were snap‐frozen in Tissue‐Tek optimum cutting temperature (OCT) compound (Sakura Finetek USA Inc., Torrance, CA) for cryo‐sectioning into 5 μm sections before staining for LDH activity in viable cells via precipitation of an insoluble purple‐blue formazan salt.^34^ After counterstaining with aqueous eosin, LDH‐positive cells appear dark blue. Histological slide sections were examined under a Nikon Ti‐S inverted microscope and scanned at ×4 using a slide scanner (PathScan Enabler 5, Meyer Instruments, Houston, TX). Slide scans were processed in FIJI^35^ using the ‘Freehand Line’ and ‘Freehand Selection’ tools to measure epidermal length and biopsy area, respectively. The red channel was adjusted using the ‘Colour Balance’ tool to increase contrast of the LDH stain before measuring, and the viable length and area were normalised to the total epidermal length and histological section area, respectively, of each biopsy.

### 2.7 Confocal microscopy

Biopsies were mounted in glass‐bottomed 60‐mm petri dishes (14 mm opening; MatTek, Ashland, MA) and imaged on a Zeiss 780 confocal laser scanning microscope on the GFP channel using ×5 and ×40 objectives. Zeiss Zen software was used to analyse z‐stacks and generate maximum intensity projections. For CLSM imaging of *S. aureus*, the blue Hoescht channel was used to capture tissue autofluorescence.

### 2.8 Scanning electron microscopy

The following protocol was adapted from Horton et al.^36^ Briefly, ex vivo human skin wounds were rinsed with PBS and fixed overnight in 5 ml of 1.5% glutaraldehyde in 0.1 M sodium phosphate buffer (pH 7.2) at 4°C. Samples were rinsed, treated with 1% osmium tetroxide for 1 h, and then washed again. Samples were dehydrated through a series of ethanol washes (30%–100%) followed by critical point drying (14 exchanges on low speed) and were subsequently mounted on aluminium stubs with a carbon adhesive tab and carbon paint. Silver paint was applied around the perimeter for improved conductivity. Samples were left to dry in a desiccator overnight. Following sputter coating with platinum to a thickness of 20 nm, samples were imaged in a scanning electron microscope (Zeiss LEO 1530‐VP) at 3 kV.

### 2.9 In vivo daily CHG treatment on human skin xenografted mice

To test the cytotoxicity of CHG on human skin wounds in vivo, we used an established xenografted mouse model of human skin wound healing.^37^, ^38^ All procedures on mice were approved by the University of Wisconsin Institutional Animal Care and Use Committee and the Research Animal Resource and Compliance office. Briefly, four male athymic nude mice (6–7 weeks old, Jackson Laboratory, Bar Harbour, ME) were grafted on bilateral flanks with partial thickness human skin procured from elective surgery. Eight weeks after engraftment and normalisation of skin architecture, 4 mm partial thickness wounds were created on each xenograft (2 per mouse—treatment and control). 2% CHG was applied daily on the wounds for 2 min followed by irrigating the treated wound with 1 ml PBS for three times using a pipette. Similarly, the control wounds received PBS application and irrigation. The xenografts received treatment daily for 14 days to mimic daily wound care, and digital images were obtained to document presence of infection and gross wound healing. In between daily wound treatments, the wounds were covered with Cuticerin® (Smith and Nephew, London, UK) and bandaged using 1‐inch wide CoFlex® (Andover, Salisbury, MA). On day 14, the xenografts were harvested and stained for LDH and H&E to assess cell viability and wound re‐epithelization, respectively.

### 2.10 Data and statistical analysis

Information regarding sample size and replication are described in the figure legends. All statistical analysis was performed using R.^39^ Pairwise comparisons for wound closure and tissue viability were conducted with the Wilcoxon Rank‐Sum test. Multiple comparisons and estimation of mean differences between inoculation conditions for each microbe were evaluated using a one‐way between subjects ANOVA with Tukey's Honest Significant Differences test. We used an *α* level of 0.05 for all statistical tests.

## 3 RESULTS

### 3.1 Establishment of an ex vivo wound contamination model to evaluate antimicrobial efficacy and cytotoxicity

**FIGURE 1:**
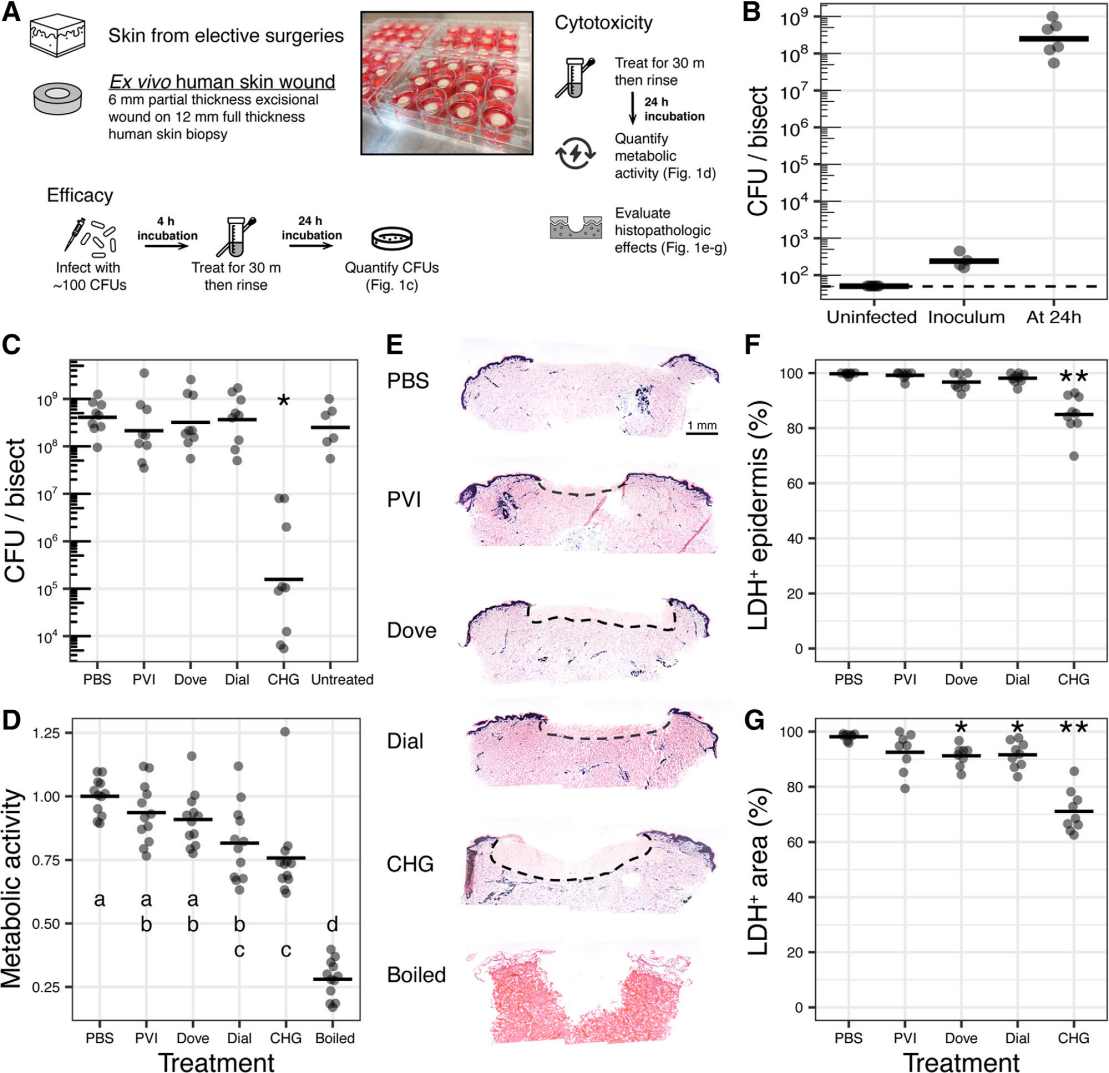
Ex vivo human excisional wound model permits dual evaluation of antiseptic efficacy and cytotoxicity. (A) Overview of model. (B) Establishment of infection with ~100 cells of *Pseudomonas aeruginosa* per 6 mm wound to represent wound contamination. Biopsies were incubated for 24 h before enumeration. Uninfected wounds had undetectable microbial growth. Horizontal bars show means of *n* ≥ 6 replicates from ≥3 skin donors. For the inoculum, *n* ≥ 4 biological replicates. (C) Quantification of viable bacterial cell counts. *N* ≥ 9 replicates (for treatments) and ≥6 replicates (for untreated controls) from ≥3 skin donors. *P adj. <0.0001. (D) Quantification of metabolic activity relative to mean of PBS treatment using the MTT assay. Boiled treatment represents non‐viable control. *N* ≥ 12 replicates from ≥4 skin donors. Treatments that do not share a common letter are significantly different from one another; *P adj*. <0.05. (E) Histopathological assessment of cellular viability using LDH staining of cryosections. Dark blue stain indicates viable cells. Dashed lines demarcate regions of depleted cellular viability. Micrographs are representative of ≥3 skin donors. (F) Quantification of viable (LDH+) epidermal length normalised to total length of epidermis. *N* ≥ 8 replicates from ≥3 skin donors. **P adj. <0.0001. (G) Quantification of viable (LDH+) area normalised to total area of histological section. *N* ≥ 8 replicates from ≥3 skin donors. *P adj. <0.05; **P adj. <0.0001.

Six mm partial thickness wounds were created on 12 mm full thickness skin biopsies cultured in individual wells of a 12‐well tissue culture plate (Figure 1A). A contamination model was developed to evaluate the activity of common clinically used wound cleansing solutions under conditions where an infection was not suspected. Each wound was inoculated with ~100 colony forming units (CFU) of *P. aeruginosa*. After 24 h of growth, 8.4 ± 0.5 (mean ± SD) log_10_ CFU/bisect of bacteria were recovered, a 6‐log increase (Figure 1B). Viable bacteria cells were not recovered above the limit of detection (50 CFU) in uninoculated wounds throughout the duration of the study (Figure 1B).

The effectiveness of five wound cleansing agents to reduce or remove bacterial bioburden was then evaluated (see Section 2.3). Cleansers were applied topically to each wound 4 h post‐inoculation and then washed after a 30 min exposure time. Quantitative viable cell counts at 24 h post‐treatment showed that CHG was the only antiseptic cleanser resulting in a significant reduction of viable bacterial counts (~3 log_10_ CFU reduction of bacterial bioburden; *P adj*. <0.0001, Figure 1C) compared with untreated control wounds.

To determine the degree of cytotoxicity for each cleanser, the ex vivo excisional wound biopsies were treated for 30 min in the absence of bacteria. Tissue metabolism was then measured 24 h post‐treatment as a surrogate for cell viability. We found that CHG and Dial® soap treatments resulted in a significant reduction of tissue metabolism compared with PBS‐treated biopsies (*P adj*. <0.05; Figure 1D). In particular, metabolism in the CHG‐treated tissue biopsies was significantly lower than that of PVI‐ and Dove®‐treated biopsies (*P adj*. <0.05; Figure 1D). Tissue viability was evaluated histologically by staining for LDH activity. A region of depleted cellular viability, identified as a loss of the dark blue staining indicative of LDH activity, was identified in the epidermis at the wound edges (Figure 1E,F) and in the mid‐reticular dermis of the wound in CHG‐treated tissue biopsies, whereas loss of cell viability was localized superficially in the dermis of the wound in soap‐treated biopsies (Figure 1E,G). Both the MTT assay and LDH staining indicate that CHG is more cytotoxic on human skin than other cleansers (Figure 1D–G). PVI did not display significant antimicrobial or cytotoxic activity compared with PBS treated control wounds.

### 3.2 Antimicrobial efficacy of CHG is transient

**FIGURE 2:**
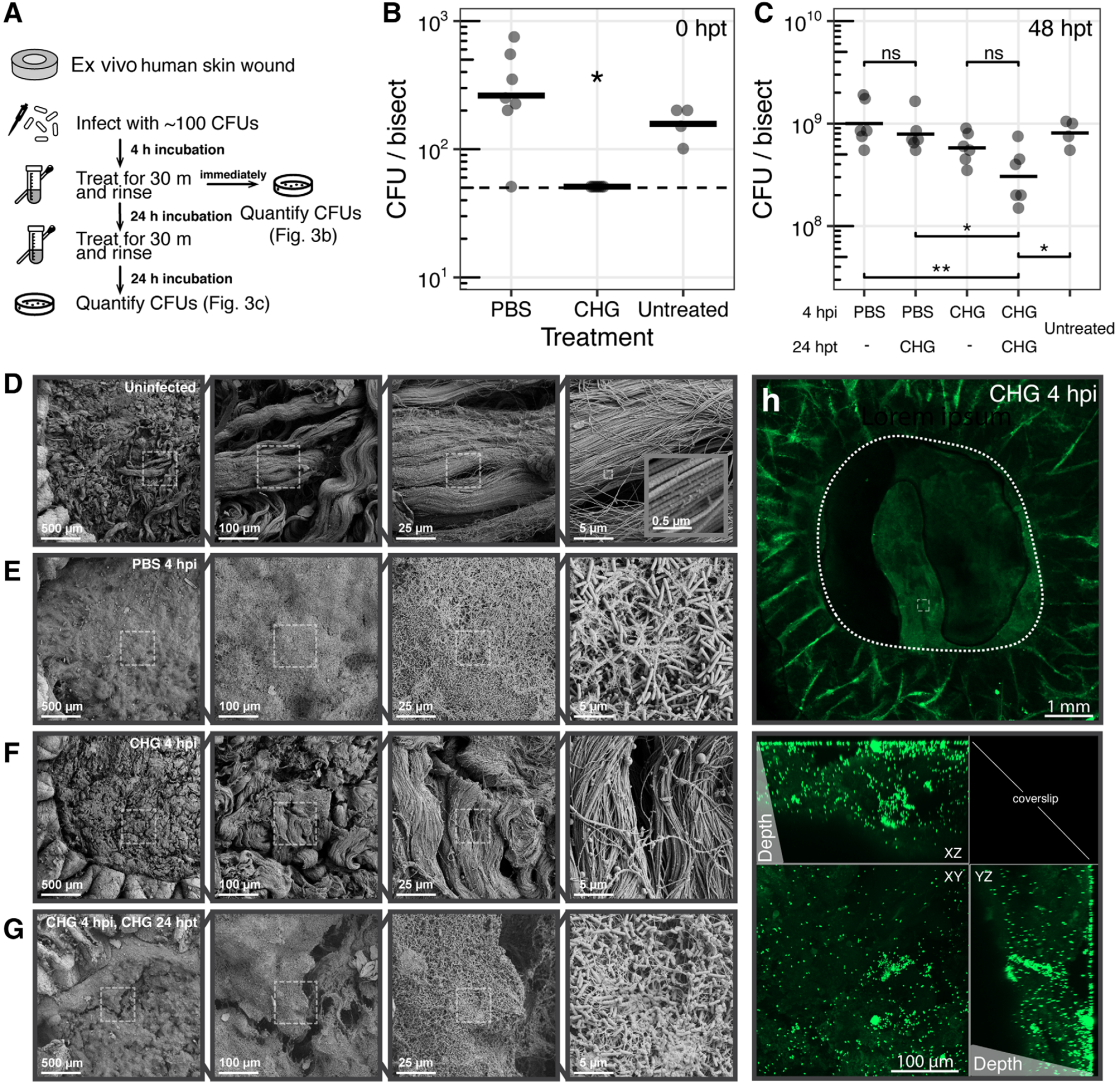
CHG antimicrobial efficacy is transient. (A) Timeline of experiments. (B) Ex vivo human excisional wound biopsies were evaluated immediately post‐treatment at 4 h post‐inoculation with ~100 cells of *P. aeruginosa*. Dashed line indicates limit of detection (50 CFU). *P adj. <0.05. (C) Total viable counts 24 h after second treatment. *N* ≥ 6 replicates (for treatments) and ≥4 replicates (for untreated controls) from ≥2 skin donors. *P adj. <0.05; **P adj. <0.01; ns, not significantly different. (D–G) Scanning electron micrographs at four different magnifications (×100, ×500, ×2000, ×10,000) of ex vivo wounds collected 24 h after final treatment. Dashed outlines represent region magnified. (D) Uninfected wound. Inset micrograph shows banding pattern of collagen fibrils at ×100,000 magnification. (E) PBS treatment at 4 h post‐inoculation. (F) CHG treatment at 4 h post‐inoculation. (G) CHG treatment at 4 h post‐inoculation and again at 24 h post‐treatment. (H) Live imaging of ex vivo wound using CLSM 24 h after CHG treatment at 4 h post‐inoculation. Circular outlines indicate the wound edge. Square outlines represent region magnified. Dark areas within the wound bed are imaging artefacts from air bubbles. Maximum intensity projection shows single cells and small aggregates of bacteria found dispersed deep within the tissue.

Since our data showed that CHG has the highest antimicrobial efficacy and the greatest cytotoxicity within a 24 h timeframe, we were interested in exploring the activity of CHG over time. We find that CHG effectively reduces viable counts to below the limit‐of‐detection (50 CFU) immediately post‐application compared with PBS treated control wounds (≤1.7 vs. 2.4 ± 0.3 log_10_ CFU/bisect; *P adj*. <0.05; Figure 2B), with some effects lasting up to 24 h post‐treatment (5.2 ± 1.2 vs. 8.6 ± 0.3 log_10_ CFU/bisect in CHG or PBS‐treated wounds, respectively; Figure 1C). However, by 48 h post‐treatment, viable cell counts increased to a level consistent with PBS‐treated wounds (8.8 ± 0.2 vs. 9.0 ± 0.2 log_10_ CFU/bisect, respectively; Figure 2C). To mimic a once‐daily clinical wound cleansing schedule, a second treatment of CHG was applied for 30 min to each contaminated wound 24 h after the first treatment, rinsed off, and incubated for another 24 h before processing. After the second CHG treatment, we observed that viable counts were not significantly different from a single CHG treatment at the same time point (8.5 ± 0.3 vs. 8.8 ± 0.2 log_10_ CFU/bisect in singly‐treated wounds; Figure 2C). CHG treatment applied 24 h after cleansing with PBS also did not result in significantly different viable counts (8.9 ± 0.2 vs. 9.0 ± 0.2 log_10_ CFU/bisect in wounds singly‐cleansed with PBS at 4 h post‐inoculation; Figure 2C).

We then used scanning electron microscopy (SEM) to qualitatively evaluate bacterial colonization and architecture of potential biofilms as well as surface topography of the wounds 24 h post‐treatment. In uninfected control wounds, the wound bed is a topologically heterogeneous substrate for microbial attachment and growth (Figure 2D). Large bundles of collagen fibres make up the connective tissue of the dermis, and are comprised of individual collagen fibrils, as shown by the clear banding pattern in the fibrils (Figure 2D, inset).^40^, ^41^ We found that colonized wounds treated with PBS at 4 h post‐inoculation were covered with a dense layer of bacteria and extracellular matrix that completely obscures the collagen fibres of the wound bed, consistent with formation of a biofilm (Figure 2E). Bacterial cells were not detected on the surface of colonized wounds treated with CHG at 4 h post‐inoculation (Figure 2F), supporting our findings that CHG is efficacious up to 24 h post‐treatment. Conversely, wounds treated with a second application of CHG at 24 h post‐treatment become covered with a dense layer of bacterial cells and extracellular matrix over the wound bed (Figure 2G), suggesting that CHG efficacy is transient and unable to suppress bacterial growth beyond the initial reduction, consistent with the quantitative culture data.

We were intrigued by the lack of visible bacteria cells on wounds treated with CHG at 4 h post‐inoculation (Figure 2F), as these wounds had a bioburden of 5.2 ± 1.2 log_10_ CFU/bisect (Figure 1C). As SEM shows only surface topology, we hypothesized that bacteria may be localized deeper in the tissue after treatment. We used confocal laser scanning microscopy (CLSM) for a depth‐resolved perspective into the tissue. This technique revealed that wounds treated with CHG at 4 h post‐inoculation contained single bacterial cells and small clusters deep within the wound bed and tissue (Figure 2H), suggesting that although bacterial cells were not detected on the surface with SEM, migration into deeper tissues results in a reservoir within the wound to repopulate the wound surface. CLSM of wounds treated with PBS at 4 h post‐inoculation and 24 h post‐treatment revealed large aggregates of *P. aeruginosa* (Figure S1A), consistent with published biofilm models of this bacterial pathogen.^42^

**FIGURE 3:**
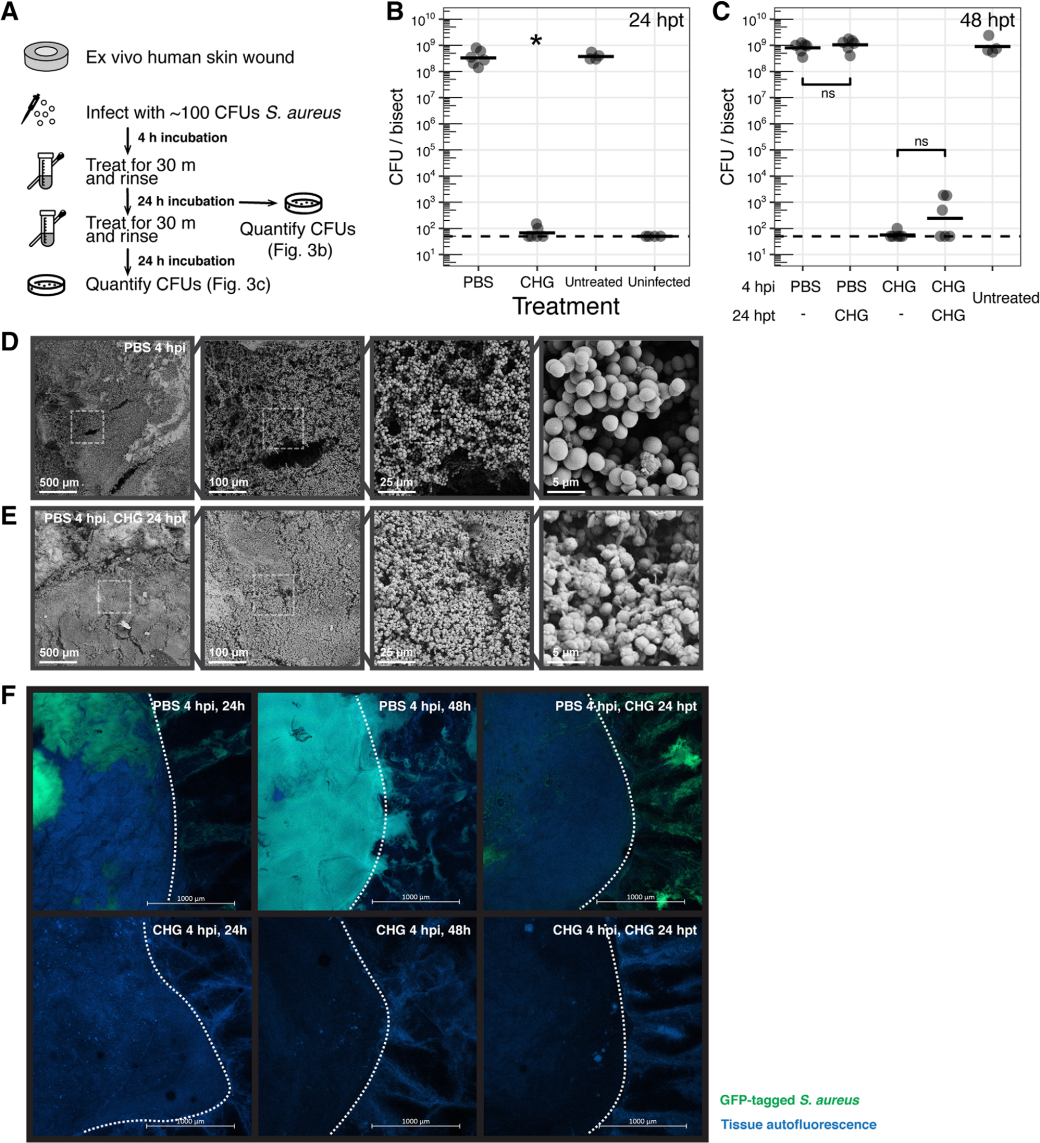
Loss of CHG efficacy against *S. aureus* biofilms. (A) Timeline of experiments. (B) Ex vivo human excisional wound biopsies were evaluated immediately post‐treatment at 4 h post‐inoculation with ~100 cells of *S. aureus*. Dashed line indicates limit of detection (50 CFU). *P adj. <0.05. (C) Total viable counts 24 h after second treatment. *N* ≥ 6 replicates (for treatments) and ≥4 replicates (for untreated controls) from ≥2 skin donors. ns, not significantly different. (D,E) Scanning electron micrographs at four different magnifications (×100, ×500, ×2000, ×10,000) of ex vivo wounds collected at 24 h after final treatment. Dashed outlines represent region magnified. (D) PBS treatment at 4 h post‐inoculation. (E) PBS treatment at 4 h post‐inoculation, CHG treatment at 24 h post‐treatment. (F) Live CLSM imaging of ex vivo wounds at low magnification 24 h after treatment. Dotted outlines indicate the wound edge. Images show GFP‐tagged *S. aureus* in the wound bed merged with blue autofluorescence of the tissue.

To determine if our results are specific to *P. aeruginosa* or Gram‐negative bacteria, we repeated the CHG treatment experiments using the contamination model with *Staphylococcus aureus*, a common Gram‐positive wound pathogen. Each wound was similarly inoculated with ~100 CFU of *S. aureus*. After 24 h of growth, 8.5 ± 0.3 log_10_ CFU/bisect of bacteria were recovered, a 6‐log increase consistent with *P. aeruginosa* (Figure 3B) growth. Treatment with CHG at 4 h post‐inoculation resulted in a significant (~6‐log CFU) reduction of bacterial bioburden at 24 h post‐treatment (*p* < 0.05, Figure 3B) compared with the PBS and untreated control. We hypothesize that the differences in bacterial response to CHG are due to the minimum inhibitory concentration of CHG against each organism which we determined to be 32 μg/ml for *P. aeruginosa* and <2 μg/ml for *S. aureus*.

Following wound bioburden suppression after a single treatment at 4 h post‐inoculation, a second CHG treatment at 24 h post‐treatment maintained lower viable counts not significantly different to a single treatment at the same time point (2.4 ± 0.8 vs. 1.7 ± 0.1 log_10_ CFU/bisect in singly‐treated wounds; Figure 3C). CHG treatment applied at 24 h after cleansing with PBS also did not result in significantly different viable counts (9.0 ± 0.2 vs. 8.9 ± 0.2 log_10_ CFU/bisect in wounds cleansed with PBS at 4 h post‐inoculation; Figure 3C), suggesting that CHG treatment is not effective against both *S. aureus* and *P. aeruginosa* once they have established within the wound tissue.

In agreement with quantitative culture data, SEM of *S. aureus* colonized wounds treated with PBS at 4 h post‐inoculation (Figure 3D) and wounds treated with a second application of CHG at 24 h post‐treatment (Figure 3E) showed dense clusters of bacterial cells and extracellular matrix over the wound bed consistent with the expected phenotype of a Staphylococcal biofilm.^43^, ^44^ This suggests CHG is not effective against biofilm phenotypes. We used CLSM at a low‐magnification to confirm binary wound bioburden phenotypes (Figure 3F). Wounds treated with PBS at 4 h post‐inoculation had visible wound and skin colonization regardless of treatment at 24 h post‐treatment (Figure 3F, top row). Conversely, wounds treated with CHG at 4 h post‐inoculation did not show detectable bacteria (Figure 3F, bottom row).

### 3.3 Cytotoxicity of CHG is long‐lasting

**FIGURE 4:**
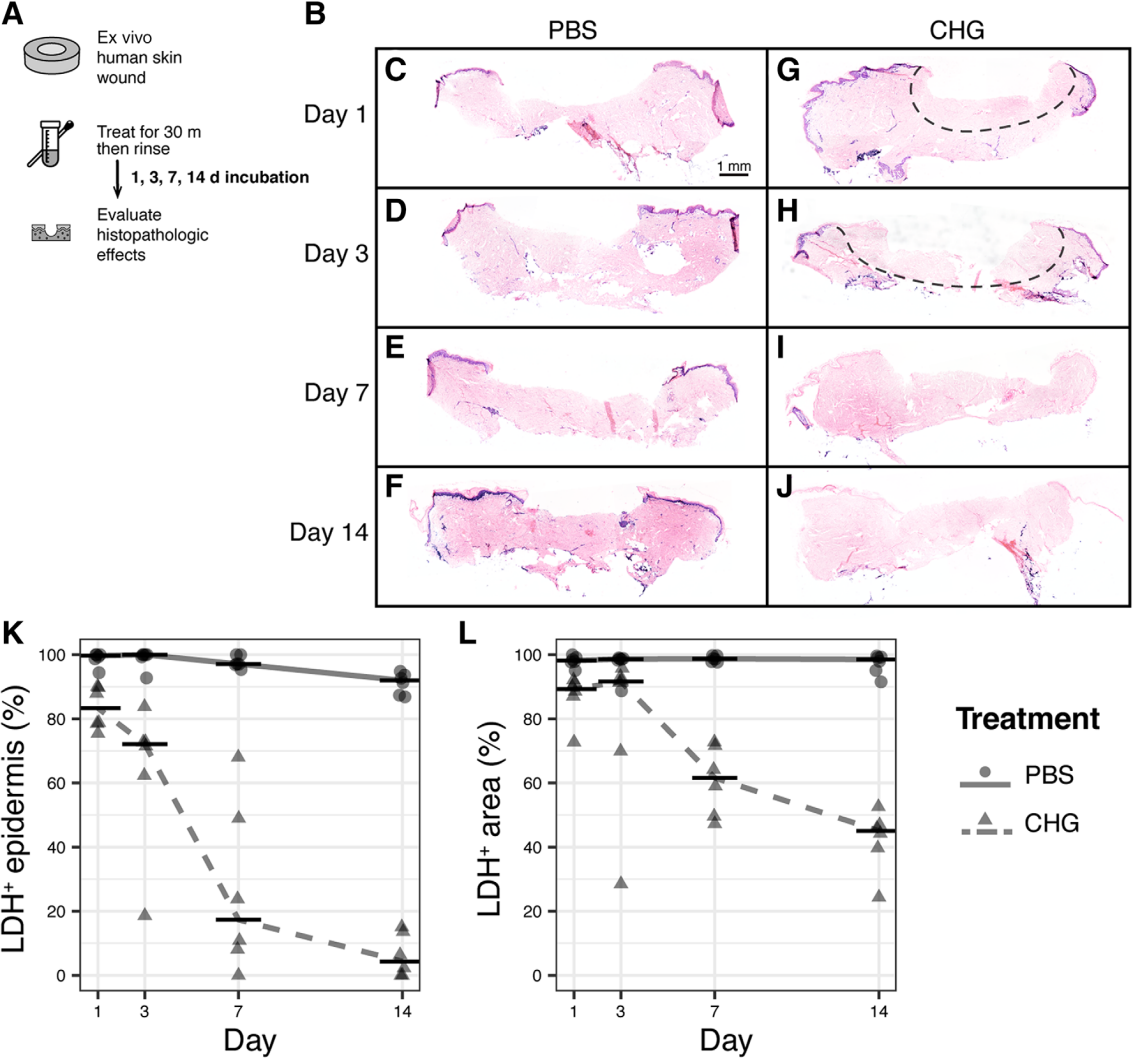
CHG exhibits progressive cytotoxicity. (A) Timeline of experiments. (B) Histopathological assessment of cellular viability using LDH staining of cryosections. Dark blue stain indicates viable cells. Dashed lines demarcate regions of depleted cellular viability. Micrographs are representative of ≥3 skin donors. (C–F) Biopsies treated with PBS and processed at 1‐, 3‐, 7‐, and 14‐days post‐treatment, respectively. (G–J) Biopsies treated with CHG and processed at 1‐, 3‐, 7‐, and 14‐days post‐treatment, respectively. (K) Quantification of viable (LDH+) epidermal length normalised to total length of epidermis. (L) Quantification of viable (LDH+) area normalised to total area of histological section. Horizontal bars show median of ≥6 replicates from ≥2 skin donors.

While we show that the antimicrobial efficacy of CHG is transient, we were interested in the long‐term cytotoxic effects of CHG in human skin. To evaluate this, we treated ex vivo wounds with either CHG or PBS for 30 min, followed by a sterile PBS rinse, and then cultured the tissue biopsies for up to 14 days (Figure 4A). At various time points, biopsies were harvested to assess cellular viability. PBS‐treated tissue biopsies remained viable for 14 days, showing dark blue staining indicative of LDH activity and cellular viability across the epidermis and throughout the dermis (Figure 4C–F,K,L). Conversely, CHG treatment resulted in a continual progression of cytotoxicity (Figure 4G–J,K,L). Consistent with earlier results (Figure 1D–G), we observed a loss of cellular viability at the epidermal wound edge and dermis where CHG was in direct contact with the tissue at 1‐day post‐treatment (Figure 4G). On day 3, this loss of cellular viability progressed laterally from the wound across the epidermis and vertically into the dermis (Figure 4H,K). By day 7, cellular viability was lost from most of the tissue (Figure 4I–L), suggesting that despite rinsing CHG after the single 30 min treatment, cytotoxicity persists and leads to a profound progression of cellular injury.

### 3.4 CHG exhibits cytotoxicity and delays wound healing in vivo

**FIGURE 5:**
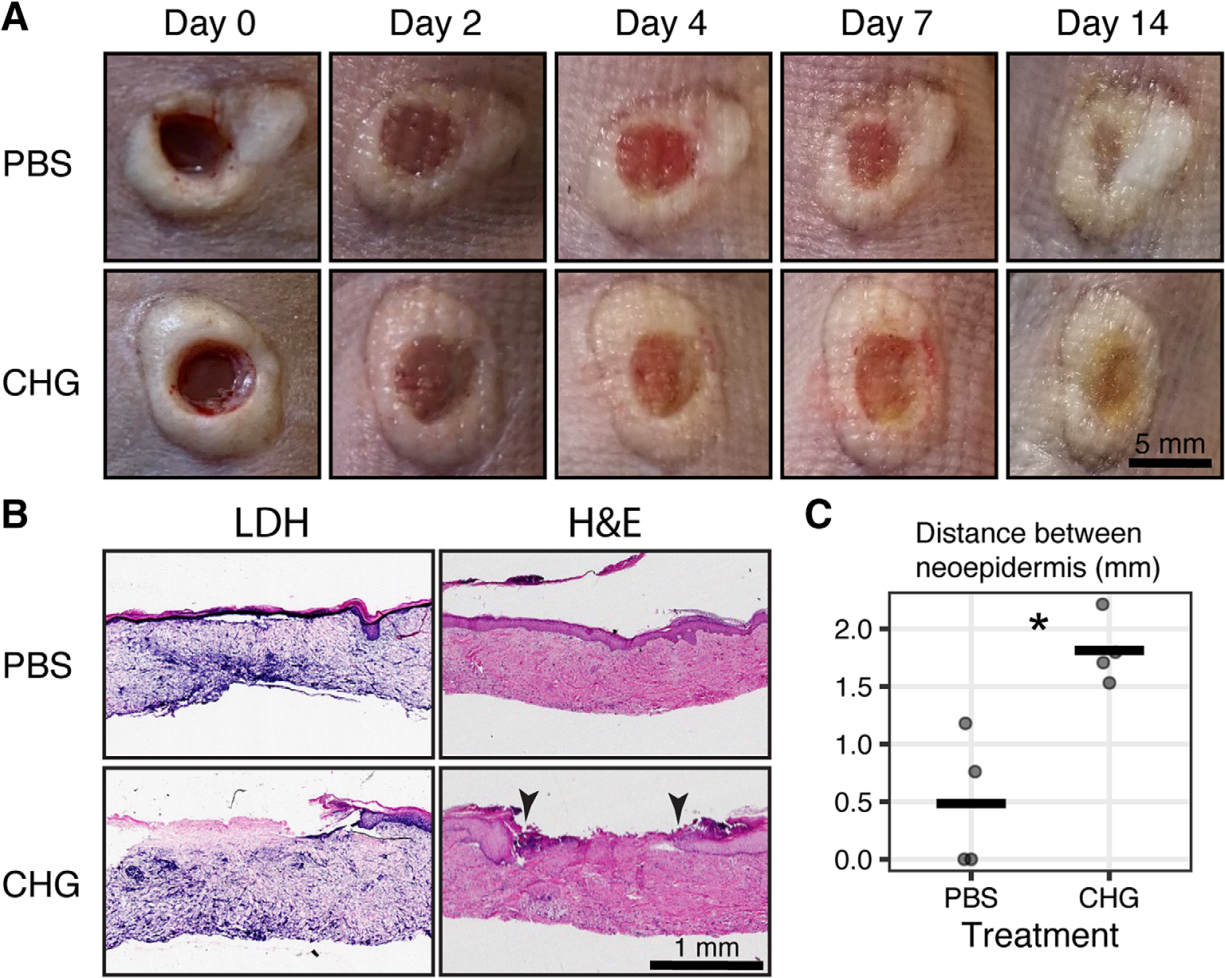
CHG is cytotoxic and impedes human skin wound healing in a human skin xenograft mouse model; 4 mm partial thickness wounds were created human skin xenografts on bilateral flanks of athymic mice. (A) Macro photographs of wounds treated with PBS and CHG daily for 14 days. (B) LDH (left column) and H&E (right column) staining of wound histological sections after 14 days of daily treatment with PBS or CHG. Arrowheads indicate unhealed wound edge in CHG‐treated wound. (D) Quantification of distances between the two epithelial tongues from H&E‐stained histological sections. *N* = 4 wounds per treatment; *N* = 4 mice; two wounds per mouse with each wound receiving a different treatment. * *p* < 0.05.

To determine if the cytotoxicity associated with CHG in ex vivo skin explants is also present in vivo where normal perfusion of the wound is present, we used a murine human skin xenograft model. Here, human skin was grafted onto the bilateral flanks of athymic mice. Eight weeks after engraftment and normalisation of skin architecture, 4 mm partial thickness wounds were created on each xenograft. To mimic a clinical wound care procedure, CHG was applied daily for 2 min followed by irrigation with PBS in the treatment wound for 14 days. The control wound received PBS application and irrigation. Macroscopic pictures of the human skin xenograft wounds over 14 days showed evidence of re‐epithelialization with minimal contraction (Figure 5A). LDH staining of histological sections collected on day 14 showed viable tissue in the PBS‐treated wounds whereas CHG‐treated wounds displayed a distinct loss of cellular viability across the epidermis and mid‐dermis (Figure 5B). H&E staining of CHG treated wounds revealed significant impairment in re‐epithelialization compared with control (Figure 5B,C), while the distance between the neo‐epidermal wound edges were significantly higher in the CHG‐treated xenograft wounds (*p* < 0.05; Figure 5C). These results replicate findings observed with daily treatment and irrigation of ex vivo wounds (Figure S2).

## 4 DISCUSSION

Wound management requires balancing strategies to reduce the risk of infection while minimizing cytotoxic effects of agents applied to wound tissue.^8^, ^45^ Here we used an ex vivo human skin excisional wound model to investigate the localization of antiseptic‐induced loss of tissue viability. We find that short exposure to chlorhexidine gluconate results in significant cytotoxicity that persists and progresses over time. We show that these effects also occur in vivo using a human skin xenograft mouse model with normal perfusion of the skin. Application of CHG to human skin engrafted on to mice leads to cellular cytotoxicity and impaired wound healing. These parallel findings in ex vivo and in vivo models support the utility of the ex vivo model as a human skin cytotoxicity testing platform. Concurrently, we demonstrate that inoculation of wound tissue with as little as ~100 bacterial cells is sufficient to allow robust growth and accumulation of microbial biomass by common wound pathogens, *Pseudomonas aeruginosa* and *Staphylococcus aureus*. While CHG imparts antiseptic activity immediately after application, it is transient and bacterial bioburden rapidly recovers after 48 h, even with serial application of CHG.

Here we elected to establish a wound contamination model using ~100 bacterial cells of common wound pathogens to evaluate antimicrobial efficacy and cytotoxicity under a more clinically relevant context with temporal resolution (Figures 1, 2, 3). This is in contrast to wound infection models that inoculate at 10^5^ CFU or higher.^46^, ^47^, ^48^ Further, classical in vitro models often expose high‐density cultures or established microbial biofilms in environmental conditions that are divorced from the host context. For example, host‐derived biofilm components such as fibrin and leukocyte‐associated proteins can alter microbial biofilm composition and structure,^30^, ^49^, ^50^, ^51^ with potential effects on antiseptic susceptibility, virulence, and pathogenesis. While a limitation of ex vivo skin models is the exclusion of the host systemic immune response as compared with in vivo animal models, the ex vivo human skin model presents a powerful tool for evaluating therapeutics while capturing the impact of the heterogeneous tissue microenvironment, such as the inter‐individual variation of cells and structures within the skin.^52^, ^53^, ^54^, ^55^, ^56^ Additionally, this model is permissive to building complexity, such as through the addition of neutrophils and other circulatory immune cells.^57^ Importantly, this model preserves the local tissue structure and heterogeneous cell population of skin, providing a clinically relevant environmental context for microbial growth.

Classical models to evaluate cytotoxicity are typically performed in vitro and also lack structural components and cellular polarization of full‐thickness skin.^17^, ^18^, ^22^, ^58^ We found that CHG displayed significant reduction in metabolic activity of the tissue, while soaps resulted in localized and progressive cytotoxicity on the wound bed (Figure 1C,D). The impact of CHG on the epidermal cells at the wound edge appears irreversible and cell death progresses both across the epidermis lateral to, and deep into, the treated wound (Figure 4). Importantly, these findings were replicated with in vivo application of CHG in a human xenograft mouse model, resulting in both local cytotoxicity and impaired wound healing compared with wounds exposed to PBS (Figure 5). We hypothesize that CHG cytotoxicity is affected by contact and diffusion gradients of the antiseptic that occur throughout the heterogeneous structure of a wound, which may also impact antiseptic efficacy on contaminating microbes. Given the ubiquitous use of CHG in clinical practice, these findings warrant further study to determine if a change in practice of CHG in wound care and in preoperative surgical preparations is necessary to prevent deleterious effects on wound healing.

Wound cleansers and antiseptic agents are often used to reduce the risk of infection and manage microbial bioburden. Here we show that CHG application is efficacious when a low bioburden of bacteria is present, such as in the case of contaminated wound. However, over time the bacterial populations of *P. aeruginosa* rise to an average of ~10^5^ CFU/bisect by 24 h post‐treatment (Figure 1C). Although the quantity of *P. aeruginosa* counts at 24 h post‐treatment remains significantly lower than PBS treated wounds, these data suggest that within our model, colonization with less than 50 CFU (the limit of detection) is permissive to allow infection to proceed. By the time bacterial bioburden reaches ~10^5^ CFU, a second application of CHG, mimicking a clinical treatment schedule, does not exert a measurable effect on microbial growth. We observed the same trend for *S. aureus* (Figure 3C) and hypothesize this is likely due to reduced antiseptic efficacy against microbial biofilms that may be forming in the wound environment.^47^ In contrast, in vitro biofilms and planktonic cells have greater susceptibility to CHG.^19^, ^47^, ^59^ Notably, PVI treatment did not lower wound bioburden, despite robust in vitro efficacy against *P. aeruginosa*.^9^, ^47^, ^60^, ^61^

Using SEM, we showed extensive surface colonization of the wound bed corresponding to high bacterial bioburden (Figures 2E and 3D). We observed a dense biofilm composed of bacterial cells and extracellular matrix, which matured into mushroom‐like aggregates for *P. aeruginosa* (Figure S1). The spatial and physical structure of microbial biofilms are important for their virulence and pathogenesis,^62^, ^63^ suggesting that physical disruption from debridement or irrigation may be synergistic to antiseptic treatment. Interestingly, wounds treated with CHG at 4 h post‐inoculation of *P. aeruginosa* showed no visible bacterial cells on the surface of the wound bed using SEM (Figure 2F). However, CLSM revealed single bacterial cells and small aggregates dispersed within the tissue that may act as a reservoir (Figure 2H). This model also allows for evaluating spatial heterogeneity of microbes within the wound. Low‐magnification CLSM of *S. aureus* inoculated wounds showed differential pockets and clusters of bacterial growth despite consistent bacterial counts (Figure 3B,C,F). Further, *P. aeruginosa* has been reported to reside deep within patient wound tissue as compared with other wound pathogens such as *S. aureus* that reside closer to the surface.^64^ We envision future studies will integrate polymicrobial interactions and spatial ecology alongside antiseptic efficacy and cytotoxicity.

In conclusion, we present a clinically relevant model for evaluating antiseptic cytotoxicity and efficacy, with the ability to resolve spatial localization and temporal dynamics of tissue viability and microbial growth. We find that the common wound antiseptic CHG displays concerning levels of cytotoxicity while antimicrobial efficacy is transient. In light of recent studies suggesting that CHG may not impact surgical site infection as previously reported and implemented in widespread guidelines for preoperative care,^13^, ^14^ our findings should raise concern about the ubiquitous use of CHG as a preoperative surgical treatment and cleanser. We anticipate that this model will bolster basic, translational, and pre‐clinical studies in wound care by providing further insights into the complex interplay between host responses and microbial growth dynamics in the context of advanced wound care.

## AUTHOR CONTRIBUTIONS

Conceptualization: J. Z. Alex Cheong, Aiping Liu, Lindsay R. Kalan, Angela L. F. Gibson. Data Curation: J. Z. Alex Cheong, Aiping Liu, Clayton J. Rust, Collin L. Tran, Sameeha E. Hassan, Lindsay R. Kalan, Angela L. F. Gibson. Formal Analysis: J. Z. Alex Cheong, Aiping Liu, Clayton J. Rust, Collin L. Tran, Sameeha E. Hassan, Lindsay R. Kalan, Angela L. F. Gibson. Supervision: Lindsay R. Kalan, Angela L. F. Gibson. Writing ‐ original draft: J. Z. Alex Cheong, Aiping Liu, Lindsay R. Kalan, Angela L. F. Gibson. Writing ‐ review & editing: J. Z. Alex Cheong, Aiping Liu, Clayton J. Rust, Collin L. Tran, Sameeha E. Hassan, Lindsay R. Kalan, Angela L. F. Gibson.

## FUNDING INFORMATION

This work was supported by grants from the Wisconsin Partnership Program [A.L.F.G.], Shapiro Summer Research Program from the School of Medicine and Public Health, University of Wisconsin–Madison [A.L.F.G.] and the National Institutes of Health (NIDDK T35 DK062709 [C.J.R., A.L.F.G.] and NIGMS R35 GM137828 [L.R.K.]).

## CONFLICT OF INTEREST

The authors state no conflict of interest.

## Supporting information


**Figure S1** *Pseudomonas aeruginosa* forms aggregates in ex vivo wounds. Live imaging of ex vivo wounds using CLSM at 24 h after PBS treatment at 4 h post‐inoculation and PBS treatment at 24 h post‐treatment. Maximum intensity projection shows a large aggregate of GFP‐expressing *Pseudomonas aeruginosa*.

**Figure S2** Daily CHG treatment exhibits cytotoxicity in ex vivo wounds
(a) LDH staining of wound histological sections after 14 days of daily treatment with PBS or CHG. (b) Quantification of metabolic activity relative to mean of PBS treatment using the MTT assay. Horizontal bars show medians of six replicates from two skin donors. *p < 0.05.

## Data Availability

Data supporting the findings of this study are available within the article and its supplementary materials. Raw data are available from the corresponding author upon reasonable request.
