# Supplementary figures and images for: Robbing Peter to Pay Paul: Chlorhexidine gluconate demonstrates short‐term efficacy and long‐term cytotoxicity
Source: Wound Repair Regen. 2022 Aug 11;30(5):573–84. doi: 10.1111/wrr.13044 (PMC9542784; doi:10.1111/wrr.13044)

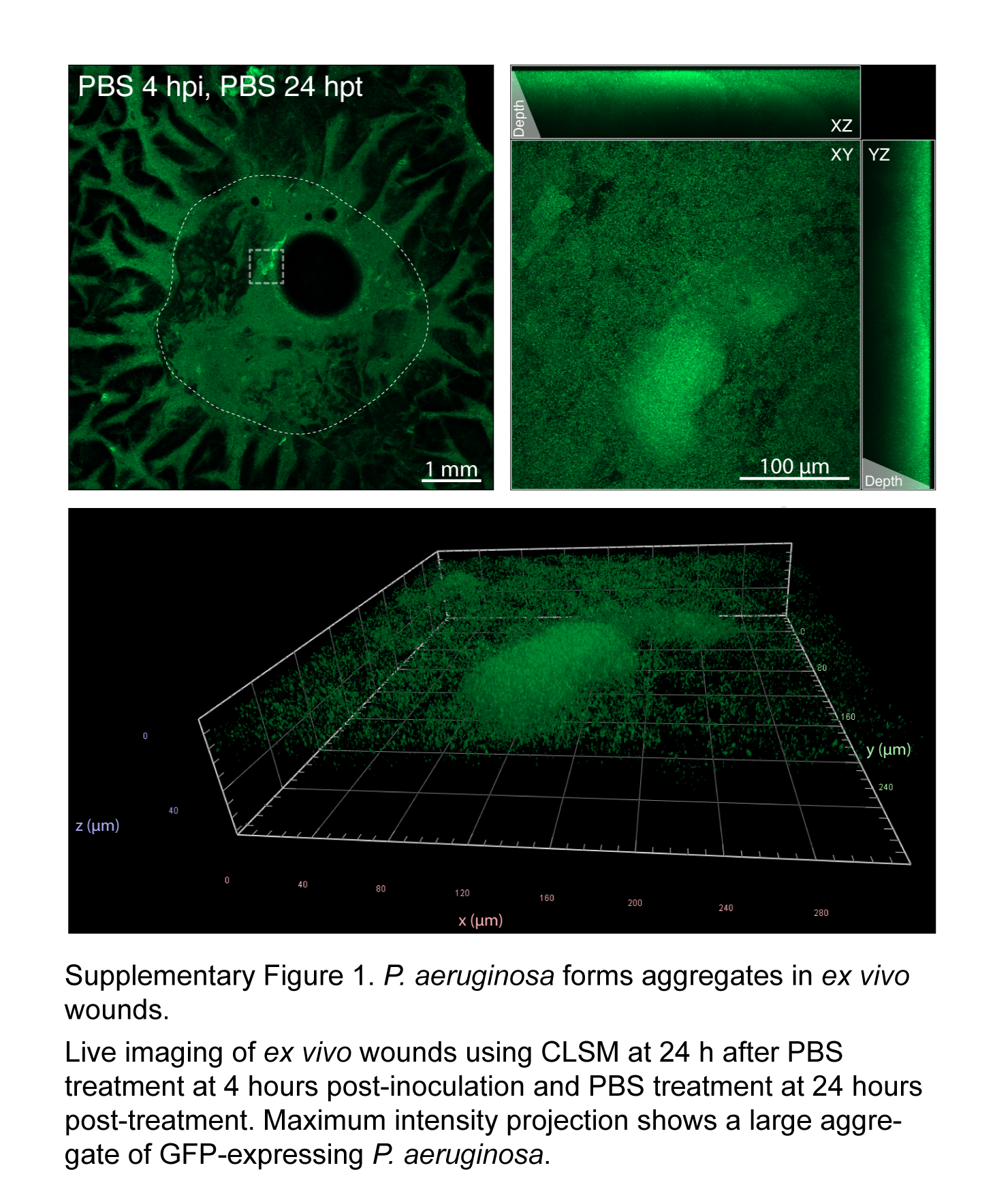

Supplement: Supplementary file 1 — Figure S1 Pseudomonas aeruginosa forms aggregates in ex vivo wounds. Live imaging of ex vivo wounds using CLSM at 24 h after PBS treatment at 4 h post‐inoculation and PBS treatment at 24 h post‐treatment. Maximum intensity projection shows a large aggregate of GFP‐expressing Pseudomonas aeruginosa. [file WRR-30-573-s002.tif]

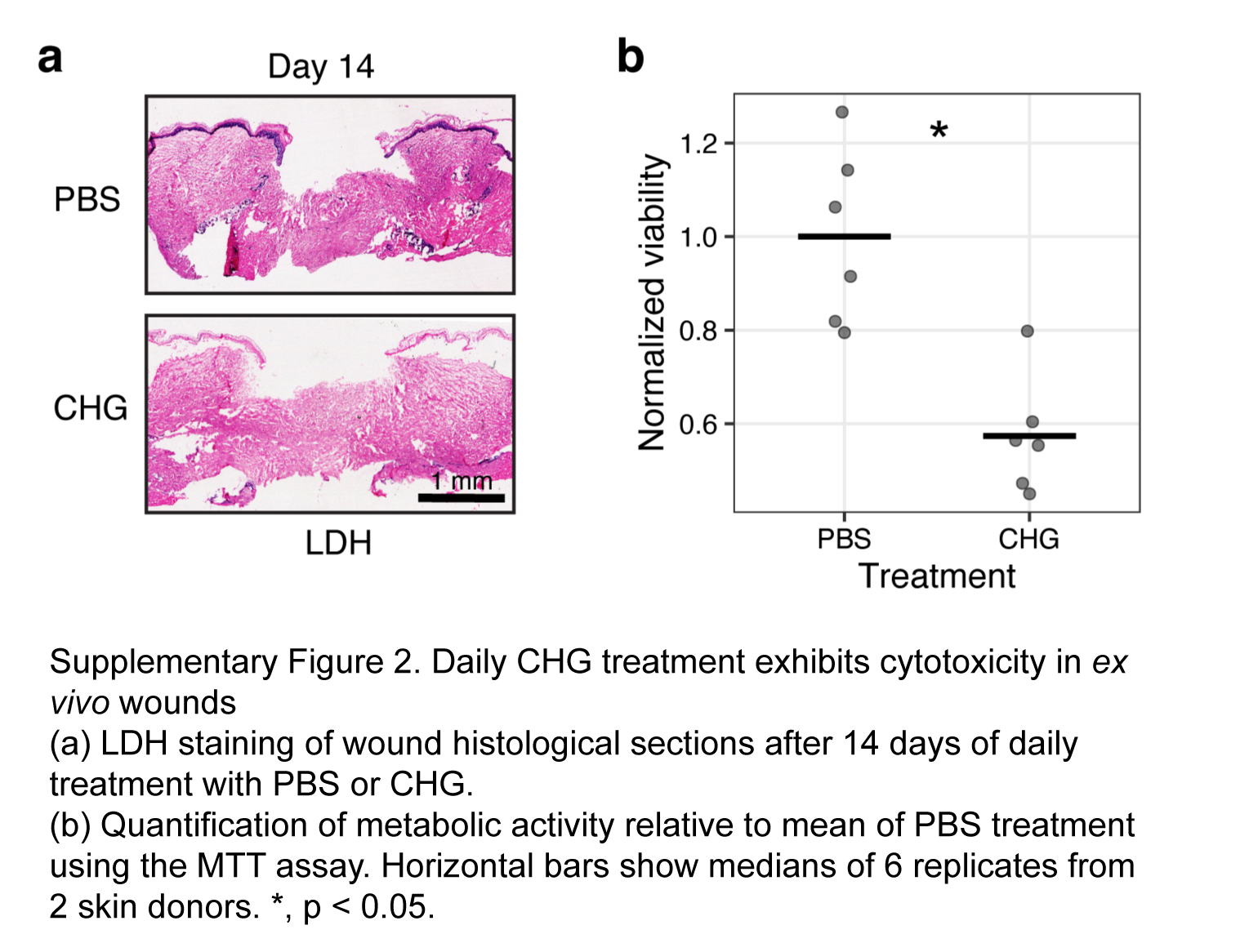

Supplement: Supplementary file 2 — Figure S2 Daily CHG treatment exhibits cytotoxicity in ex vivo wounds (a) LDH staining of wound histological sections after 14 days of daily treatment with PBS or CHG. (b) Quantification of metabolic activity relative to mean of PBS treatment using the MTT assay. Horizontal bars show medians of six replicates from two skin donors. *p < 0.05. [file WRR-30-573-s001.tif]
